# The disulfide bond Cys2724-Cys2774 in the C-terminal cystine knot domain of von Willebrand factor is critical for its dimerization and secretion

**DOI:** 10.1186/s12959-021-00348-w

**Published:** 2021-11-27

**Authors:** Yuxin Zhang, Fengwu Chen, Aizhen Yang, Xiaoying Wang, Yue Han, Depei Wu, Yi Wu, Jingyu Zhang

**Affiliations:** 1 Department of Hematology, Key Laboratory of Hematology of Hebei Province, The Second Hospital of Hebei Medical University, 050000 Shijiazhuang, China; 2 National Clinical Research Center for Hematologic Diseases, the First Affiliated Hospital, Collaborative Innovation Center of Hematology, State Key Laboratory of Radiation Medicine and Prevention, Cyrus Tang Medical Institute, Soochow University, 215123 Suzhou, China

**Keywords:** von Willebrand factor (VWF), von Willebrand disease (VWD), cystine knot domain, multimerization, disulfide bond

## Abstract

**Background:**

Type 3 von Willebrand disease (VWD) exhibits severe hemorrhagic tendency with complicated pathogenesis. The C-terminal cystine knot (CTCK) domain plays an important role in the dimerization and secretion of von Willebrand factor (VWF). The CTCK domain has four intrachain disulfide bonds including Cys2724-Cys2774, Cys2739-Cys2788, Cys2750-Cys2804 and Cys2754-Cys2806, and the single cysteine mutation in Cys2739-Cys2788, Cys2750-Cys2804 and Cys2754-Cys2806 result in type 3 VWD, demonstrating the crucial role of these three disulfide bonds in VWF biosynthesis, however, the role of the remaining disulfide bond Cys2724-Cys2774 remains unclear.

**Method and results:**

In this study, by the next-generation sequencing we found a missense mutation a c.8171G>A (C2724Y) in the CTCK domain of *VWF* allele in a patient family with type 3 VWD. In vitro, VWF C2724Y protein was expressed normally in HEK-293T cells but did not form a dimer or secrete into cell culture medium, suggesting that C2724 is critical for the VWF dimerization, and thus for VWF multimerization and secretion.

**Conclusions:**

Our findings provide the first genetic evidence for the important role of Cys2724-Cys2774 in VWF biosynthesis and secretion. Therefore, all of the four intrachain disulfide bonds in CTCK monomer contribute to VWF dimerization and secretion.

## Introduction

VWF is a large-molecular multimerized glycoprotein synthesized and secreted by endothelial cells and megakaryocytes. This protein has multiple contributions to the hemostatic process: it mediates platelet adhesion and aggregation at sites of vascular injury and carries coagulation factor VIII (FVIII) in the circulation[1]. Functional and/or quantitative deficiencies of VWF are known as von Willebrand disease (VWD), a disorder affecting 0.01–1% of the population[1]. These patients with VWF deficiency manifest a severe hemorrhagic phenotype, originating from defective formation of platelet-rich thrombi and a secondary deficiency of FVIII impairing the generation of a fibrin network[1]. Quantitative deficiencies of VWF result from changes in biosynthesis, secretion, and/or clearance of the protein.

VWF processor contains multiple domains in the order of D1-D2-D9-D3-A1-A2-A3D4-B1-B2-B3-C1-C2-CK[2]. The VWF sequence contains an unusually high content of cysteine residues (8.3%), a percentage fourfold higher than the average in human proteins. In the biosynthesis of VWF, its multimerization is a critical step and involves the pairing of cysteines. pro-VWF monomers are linked pairwise through interchain disulfide bond(s) at the C-terminal cystine knot (CTCK) domain[3]. The CTCK domain has 11 cysteines, 8 of which form 4 intrachain disulfide bonds, Cys2724-Cys2774, Cys2739-Cys2788, Cys2750-Cys2804 and Cys2754-Cys2806, the other 3 cysteines form 3 interchain disulfide bonds [4] ensuring the long-term stability of the dimeric conformation of VWF. In the endoplasmic reticulum (ER), pro-VWF subunits first engage into a covalent connection, involving the formation of 3 interchain cysteine pairs located in the C-terminal cysteine knot (CK) domains. Importantly, the structural location of these 3 intermolecular pairs protects them from disulfide reduction, ensuring the long-term stability of the dimeric conformation of VWF. Homozygous mutations of C2739Y[5], C2804Y[6], C2754W[7, 8], and C2806R[9] caused type 3 VWD. These mutations impair C-terminal dimerization and consequently, preventing the formation of long multimers, demonstrating that these three disulfide bonds Cys2739-Cys2788, Cys2750-Cys2804, and Cys2754-Cys2806 are critical for VWF biosynthesis. However, the functional role of the remaining disulfide bond of Cys2724-Cys2774 in VWF biosynthesis remains unknown.

In this study, we diagnosed a patient family with type 3 VWD, next-generation sequencing revealed that the proband had a *VWF* allele with a c.3814delT deletion mutation and the other *VWF* allele with a c.8171G>A (C2724Y) missense mutation in CK domain. In a cell expression system, we found that the VWF C2724Y mutation did not affect VWF monomer expression but caused defective VWF dimerization and secretion. These data provide the first evidence for the critical role of the disulfide bond Cys2724-Cys2774 in CTCK domain in VWF biosynthesis and secretion.

## Materials and methods

### Blood samples and laboratory tests

This study was approved by the Ethics Committee of the Second Hospital of Hebei Medical University. All patients were informed about the experimental nature of this study and gave their consent to participation. Venous blood collected using 105 mM sodium citrate as anti-coagulant was centrifuged at 4000 g for 10 min at room temperature (RT), followed by collection of plasma. Within 2 h after sample collection, activated partial thromboplastin time (APTT) and FVIII coagulant activities (FVIII:C) were tested by an automatic coagulation instrument (ACLTOP700, Spanish Wolfen Group). Plasma VWF antigen (VWF:Ag) was measured using an enzyme-linked immunosorbent assay (ELISA) kit according to the manufacturer’s instructions (HemosIL® von Willebrand Factor Antigen, Spanish Wolfen Group).

### VWF multimer analysis

Analysis of VWF multimers in plasma from patients, cell lysate, and cell cultural medium was performed using 1.5% SDS-agarose gel electrophoresis, as previously described with slight modification[10, 11]. Briefly, the plasma was diluted 1: 5 and cell lysate and cultural medium were diluted 1: 3 with sample dilution buffer (0.5 M Tris/HCl, pH 6.8, 0.5 M EDTA, 0.1% SDS, 9 M Urea) and heated at 60 °C for 30 minutes. Fifteen µL of sample was loaded and VWF multimer was separated by electrophoresis at 3 mA for 10 h. The gel was washed with double distilled (dd) H_2_O, air-dried and blocked with 5% skimmed milk. VWF multimers were detected by immunoblotting with a rabbit anti-human VWF antibody (Dako).

### Screening for mutations

Genomic DNA of the blood cells from the proband was screened by next-generation sequencing. Two mutations c.3814delT deletion and c.8171G>A mutation in *VWF* gene of the proband were discovered, the first-generation sequencing was used to verify the mutations. DNA sequences containing the c.3814delT deletion mutation and c.8171G>A mutation in exon 28 and exon 52 of the *VWF* gene were amplified by polymerase chain reaction (PCR) using the primers of VWF-6059034-F: 5’- GGATAGGTATCCGAACACGGAG-3’, VWF-6,059,034-R: 5’- ACAAGAGGGTTGCTTTAGCCAT-3’, VWF-6,128,770-F: 5’- CAGCTCTGACGGTCGCTTC-3’, and VWF-6,128,770-R: 5’- TCTGTGGGAATATGGAAGTCATTG-3’. The amplified DNA fragments were sequenced directly.

### Plasmid construction

The plasmid pSVHVWF containing the full-length wild-type human *VWF* cDNA was kindly provided by Dr. Evan Sadler (Washington University School of Medicine, St Louis, USA). Plasmid of VWF C2724Y was constructed by PCR-based mutagenesis using the pSVHVWF plasmid expressing VWF-WT (wild-type) as a template. The sequence of site-directed mutation primers is 5’- GTGAGGAGCCTGAGTACAACGACATCACTGC-3’. PCR amplification conditions: pre-denaturation at 95℃ for 30 s; denaturation at 98℃ for 10 s, annealing at 65℃ for 30 s, extension at 72℃ for 10 min, repair extension at 72℃ for 15 min after 22 cycles. PCR products were digested at 37℃ by the enzyme of Dpn I for 3 h and transformed into *E. coli* DH5α competent cells by the method of heat shock. Plasmids of VWF WT and VWF C2724Y were purified with the endotoxin-free plasmid extraction kit from Qiagen, and verified by sequencing.

### Expression of recombinant VWF protein in HEK-293T cells

HEK-293T cells were cultured in Dulbecco’s modified Eagle’s medium (DMEM) with high glucose supplemented with 10% fetal bovine serum. Cells were seeded in 6well plate to reach 50-70% confluence at transfection. Transient transfection was performed with a total of 2 µg of WT or C2427Y mutant VWF plasmid, or mixture of WT and C2427Y mutant plasmids using PolyJet™ transfection reagent according to the manufacturer’s instructions (SignaGen^@^ Laboratories). After 48 h, cells and culture supernatant were harvested, and the cells were lysed with a buffer containing 100 mM Tris/HCl, pH 7.5, 1% Triton X-100 and proteinase inhibitor cocktail (Sigma). Cell lysate and culture supernatant was concentrated to 1/10 of the starting volume using an ultrafiltration cassette (Millipore), and analyzed for VWF expression and multimerization.

## Results

### Diagnosis of a patient family with type 3 VWD

**Fig. 1:**
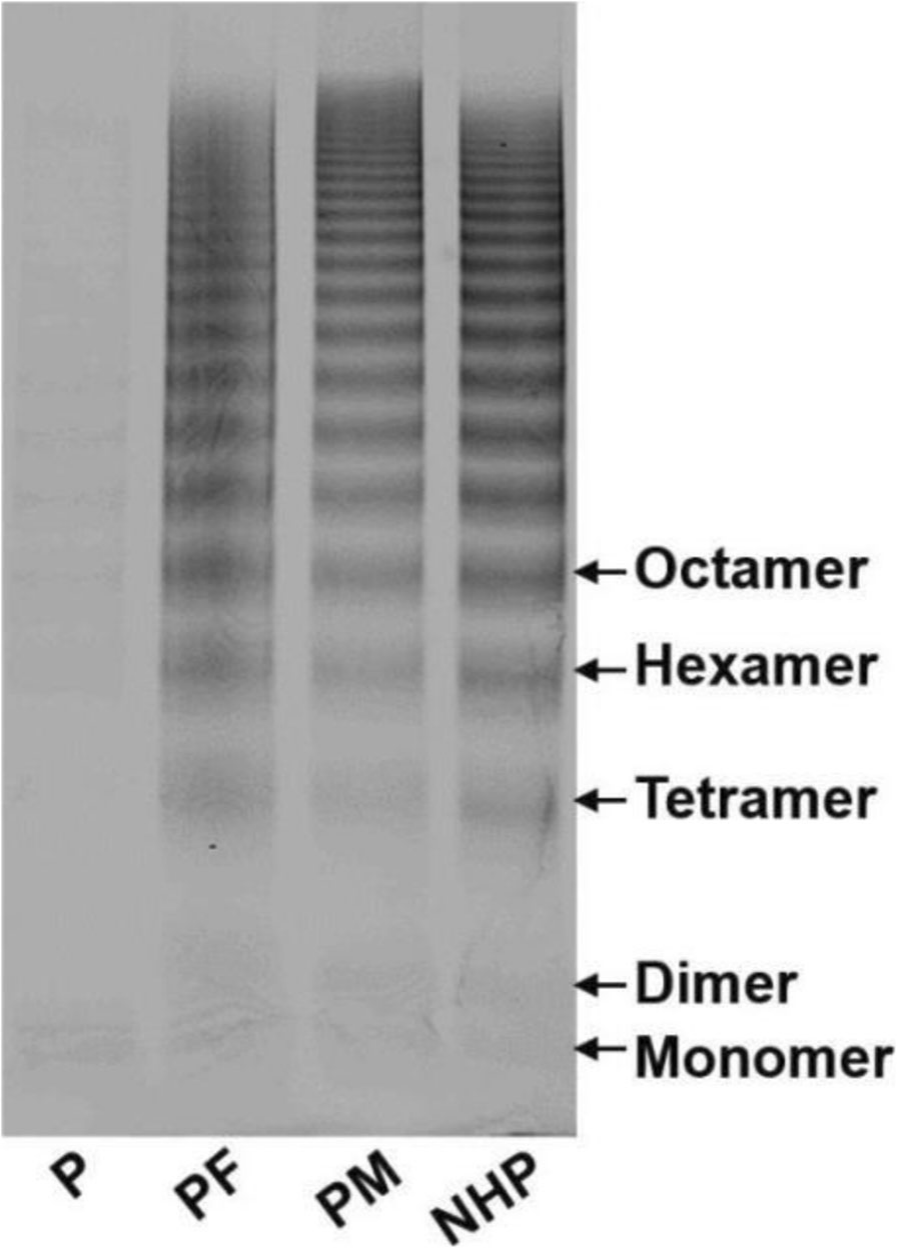
VWF multimer analysis of plasma from the family with type 3 VWD. Plasma VWF multimers were assessed by 1.5% SDS-agarose gel non-reducing electrophoresis and Western blotting. All plasma samples were diluted 1:5 with sample dilution buffer. NHP, normal human plasma; P, proband; PF, proband’s father; PM, proband’s mother

The proband was a 12-year-old girl, suffered frequently from large ecchymosis in bump injury since childhood. She was diagnosed as type 3 VWD for intermittent petechiae and coagulopathy when she was 5 years old. She had a history of admitting to hospital with severe menorrhagia and was treated with transfusion of cryoprecipitated FVIII when she was 11 years old. During the next year, she experienced several times of excessive menstrual bleeding. Neither her parents nor her sister at 20-year-old had a bleeding tendency. The results of laboratory tests showed that the proband had a prolonged activated partial thromboplastin time (APTT, 66.8 s), low plasma VWF Antigen level (VWF:Ag, 2%) and markedly reduced FVIII coagulant activities (FVIII:C, 2%). As shown in Fig. 1, the multimer analysis of plasma VWF showed that the VWF multimer was almost missing in the proband, while VWF from the proband’s parents had a normal concentration and multimer distribution. This result explained the abnormal coagulation activity of the plasma from the proband and was consistent with the diagnosis of type 3 VWD.

### Identification of candidate VWF gene mutations

**Fig. 2:**
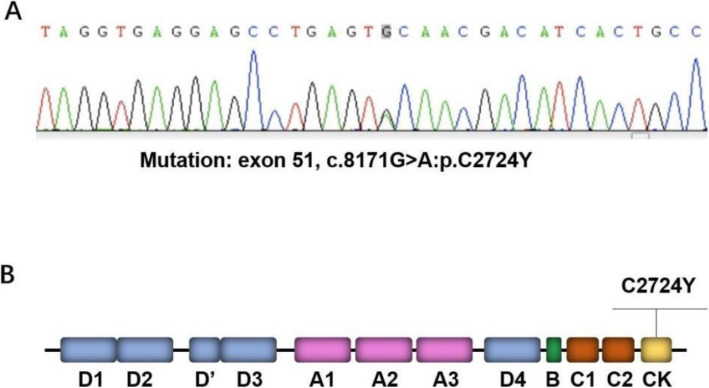
Identification of candidate VWF gene mutations. **A** Results of first-generation sequencing showed that one *VWF* allele of the proband had a c.8171G>A:p.C2724Y mutation in exon 51. **B** The location of the C2724Y mutation site in the CK domain of VWF molecular

To understand the genetic basis underlying the VWD in this family, we isolated genomic DNA from the blood cells of the proband, with which the next-generation genetic sequencing was performed. Sequencing analysis revealed that the proband had a *VWF* allele from her mother with a c.3814delT deletion mutation in exon 28, predicting a frameshift-mutations involving the loss of Cys1272; and the other *VWF* allele from her father had a c.8171G>A missense mutation in exon 51, predicting a substitution of cysteine with tyrosine at amino acid 2724. The accuracy of the results of next-generation genetic sequencing was verified by the first-generation sequencing (Fig. 2 A). Cys2724 locates in the CK domain (Fig. 2B).

### Expression of recombinant WT and mutant VWF in mammalian cells

**Fig. 3:**
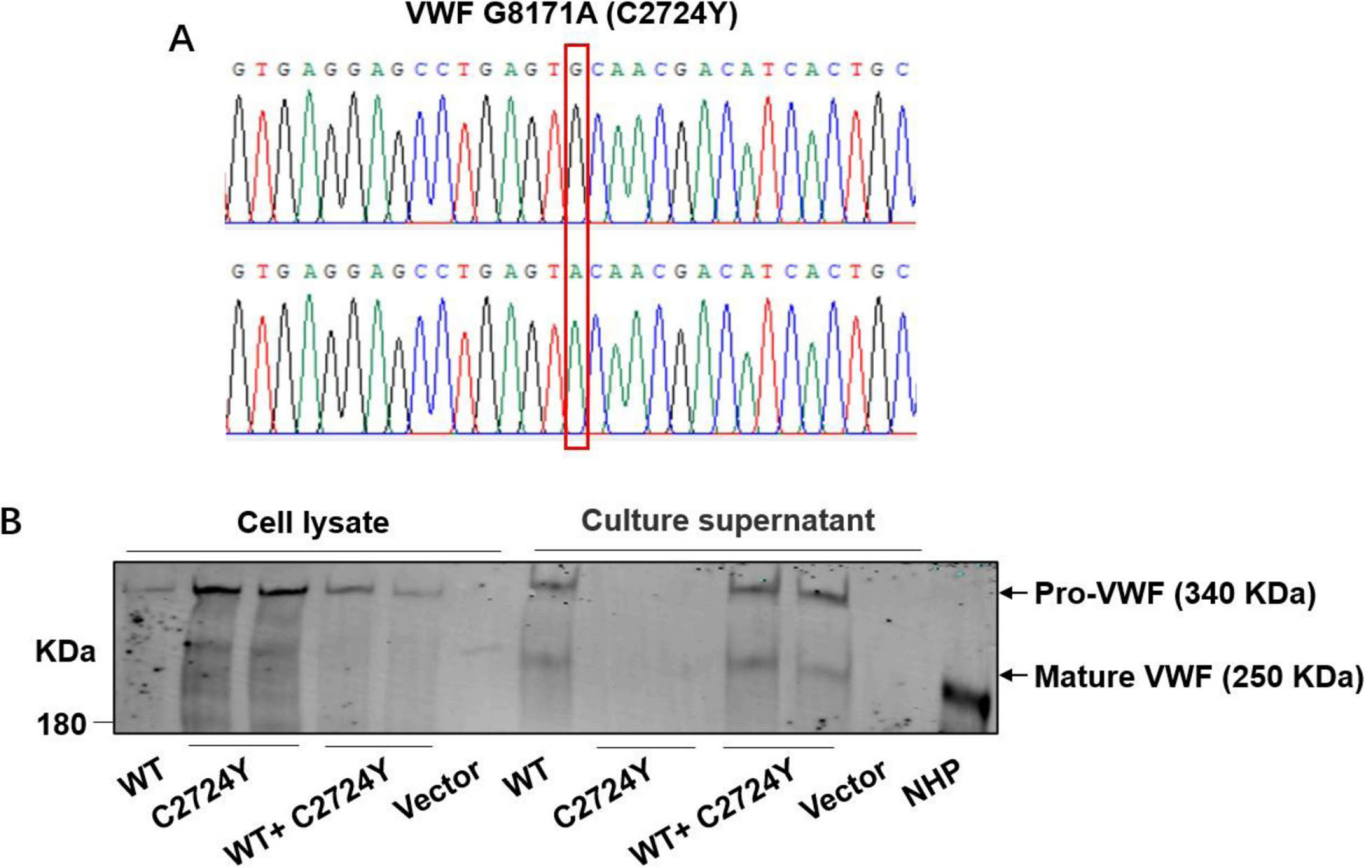
Expression of recombinant VWF protein in HEK-293T cells.** A** The results of genetic sequencing showed a G >A mutation at position 8171 of VWF. **B** Western blotting analysis of concentrated lysate and supernatant from cultured cells transfected with the plasmids of VWF WT, VWF C2724Y, and mixture of VWF WT and VWF C2724Y. Protein in the lysate and supernatant was separated by 8% reducing SDS-PAGE and immunoblotted with anti-VWF antibody

To determine the mechanism of C2724Y mutation leading to type 3 VWD, we constructed a C2724Y mutant *VWF* plasmid by PCR-based site-directed mutagenesis. The results of genetic sequencing indicated a successful mutation of Cys2724 in VWF cDNA (Fig. 3 A). To characterize the effect of the C2724Y mutation on VWF synthesis and secretion, HEK-293T cells were transfected with plasmids of VWFWT, VWF-C2724Y, and mixture of VWF-WT and VWF-C2724Y. After 72 h, the transfected cells and culture supernatant was tested for VWF antigen level by reduced SDS-PAGE and western blotting. As shown in Fig. 3B, recombinant VWF C2724Y protein was expressed normally in HEK-293T cells, but it could not be secreted into cell culture medium, compared with VWF-WT protein. This result, which is consistent with the reduced VWF antigen level in plasma of the proband with the C2724Y mutant, suggests that C2724-C2774 disulfide bond is critical for the VWF secretion.

### Multimer analysis of recombinant VWF-C2724Y protein expressed in HEK293T cells

**Fig. 4:**
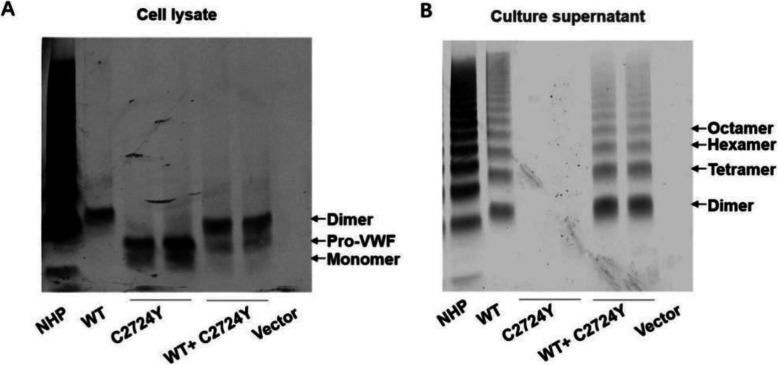
Multimer analysis of recombinant VWF protein in HEK-293T cells. Multimer analysis of concentrated lysate (**A**) and supernatant (**B**) of cultured cells transfected with the plasmid of vector, VWF WT, VWF C2724Y, and mixture of VWF WT and VWF C2724Y (1:2). Protein in the lysate and culture supernatant was separated by non-reducing electrophoresis on a 1.5% agarose gel and immunoblotted with anti-VWF antibody. NHP, normal human plasma

To further determine whether C2724Y mutation affects VWF multimerization, we analyzed the multimer pattern of the lysate and culture supernatant of cells transfected with the plasmid of VWF-WT, VWF-C2724Y, and mixture of VWF-WT and VWFC2724Y. As shown in Fig. 4 A, both recombinant VWF-WT and VWF-C2724Y were expressed in HEK-293T cell, however, dimerized product was observed only with the recombinant VWF-WT, while VWF-C2724Y only had a single band of monomer. In cell culture supernatant, there was no expression of VWF C2724Y, although normal expression and multimer pattern of VWF WT and co-expression of VWF WT and VWF C2724Y were detected (Fig. 4-B), consistent with the immunoblotting in the reduced condition. These results indicated that mutation of C2724Y causes defective VWF dimerization, which further leads to dysfunction of VWF multimerization and secretion.

## Discussion

Cys2724 has been reported to form a disulfide bond with Cys2774. In this study, we found a new type 3 VWD family with the proband having almost no VWF antigen and markedly reduced activity of VWF:RCo and FVIII:C (<5%) [12]. The proband experienced several times of severe menorrhagia, had a prolonged APTT, low level of plasma VWF Antigen (2%), and markedly reduced FVIII coagulant activities (2%). The proband had a *VWF* allele with a c.3814delT deletion mutation in exon 28, and the other *VWF* allele with a c.8171G>A missense mutation in exon 51. The latter mutation causes a substitution of cysteine 2724 to tyrosine. These data provide new evidence revealing the critical role of the disulfide bond Cys2724-Cys2774 in VWF biosynthesis.

There are 11 cysteines contained in VWF CK domain, 8 of them form 4 intrachain disulfide bonds, Cys2724-Cys2774, Cys2739-Cys2788, Cys2750-Cys2804, and Cys2754-Cys2806, the other 3 cysteines form 3 interchain disulfide bonds, Cys2771- Cys2773’, Cys2771’-Cys2773, and Cys2811-Cys2811‘[4]. Mutations of C2739Y[5], C2754W[7, 8], C2804Y[6], and C2806R[9] have also been proven to cause type 3 VWD phenotype, indicating a critical role of disulfide bonds Cys2739-Cys2788, Cys2750-Cys2804, and Cys2754-Cys2806 in VWF multimerization and secretion. Compared with the mutations of cysteines forming intrachain disulfide bonds, the mutations of cysteines in interchain disulfide bonds in CK domains did not cause serious phenotypes. Homozygous mutations of C2771S/C2771Y[13] and C2773S[14, 15] did not affect secretion of VWF protein, manifested as type 2 A VWD, subtype IID (VWD 2 A/IID). Mutation of Cys2811 affects neither VWF dimerization nor VWF secretion, but leads to the occurrence of odd-numbered multimer[15–17] (Table 1). These results indicate that the intrachain disulfide bonds in CK domain are much more important than the interchain disulfide bonds in VWF dimerization and secretion. Up to now, mutations of VWF Cys2750, Cys2774, or Cys2788 have not yet been reported.

**Table 1:** Mutations of disulfide bond in VWF CK domain reported in the literatures

	Disulfide bond	Mutation	Phenotype	Reference
Intrachain	Cys2724-Cys2774	C2724Y	Type 3 VWD	This paper
	Cys2739-Csy2788	C2739Y	Type 3 VWD	[5]
	Cys2750-Csy2804	C2804Y	Type 3 VWD	[6]
	Cys2754-Cys2806	C2754W, C2806R	Type 3 VWD	[7–9]
Interchain	Cys2771-Cys2773’	C2771S/C2771Y, C2773S	Type 2 A/IID VWD	[13–15]
	Cys2771’-Cys2773	C2771S/C2771Y, C2773S	Type 2 A/IID VWD	[13–15]
	Cys2811-Cys2811’	C2811A	Normal VWF multimerization and secretion, but occurrence of odd-numbered multimer	[15–17]

Cys2724 locates at the N-terminal of VWF CK domain, forms an intrachain disulfide bond with Cys2774[4]. In this study, we provided the evidence showing that the disruption of this Cys2724-CysC2774 caused defective VWF multimerization and secretion in cells, a potential new mechanism underlying the pathogenesis of type 3 VWD. To determine the mechanism of C2724Y mutation leading to type 3 VWD, we constructed a VWF-C2724Y plasmid, then transfected plasmid of VWF-WT, VWF-C2724Y, or together into HEK-293T cells, and analyzed the expression and multimerization of recombinant VWF in transfected cells and cultural supernatant. The results showed that recombinant VWF-C2724Y protein expressed normally in HEK-293T cells, however, it cannot form a dimer or secrete into cell culture medium, while VWF-WT protein expressed and secreted normally, as well had a normal multimer pattern (Fig. 3B). We transfected the plasmids into Hela cells and get the same results (data not shown). These results indicated that mutation of C2724Y causes defective VWF dimerization, which further leads to defective VWF multimerization and secretion, which interprets the reduction of VWF antigen level in plasma from the proband. Based on our new finding of the mutation of C2724Y in new family of type 3 VWD, all of the four intrachain disulfide bonds (Cys2724-Cys2774, Cys2739-Cys2788, Cys2750-Cys2804, and Cys2754-Cys2806) in CK monomer are critical for VWF dimerization and secretion, the disruption of any of these disulfide bonds may lead to the significant reduction of VWF antigen in plasma and development of type 3 VWD.

Although Cys2754, Cys2773, Cys2739, and Cys2724 are cysteines of interchain disulfide bonds in CK domains, and mutations of them disrupt the dimerization of VWF and result in abnormal multimerization, however, there are odd-numbered multimers in plasma from patients with heterozygous C2754W[7, 8]and C2773S[14, 15] mutations, as well as the medium of co-expression of VWF WT and C2754W or C2773S protein, while the plasma from patients with heterozygous C2724Y, and medium of co-expression of VWF WT and C2739Y[18] or C2724Y protein had no odd-numbered multimers. The underlying mechanisms remain further study. As shown in the VWF multimer analysis of cell lysate, VWF with C2739Y mutation cannot form a dimer and exist in the form of pro-VWF(Fig. 4 A), supporting that the dimer formation of pro-VWF through the N-terminus in Golgi apparatus is dependent on the formation of CK-linked dimer in ER[19].

## Conclusions

In summary, we have identified a novel missense C2724Y mutation in VWF CK domain in a family of patients with type 3 VWD. This C2724Y mutation caused defective VWF dimerization and secretion, providing the first evidence showing critical role of the disulfide bond Cys2724-Cys2774 in VWF biosynthesis.

## Data Availability

The datasets during and/or analysed during the current study available from the corresponding author on reasonable request.
